# Endoscopic and clinicopathological features of segmental colitis associated with diverticulosis

**DOI:** 10.1002/deo2.356

**Published:** 2024-04-02

**Authors:** Kazuhiko Obata, Kan Uchiyama, Ryuzo Murai

**Affiliations:** ^1^ Department of Gastroenterology Onaka‐kai Onaka Clinic Tokyo Japan; ^2^ Department of Gastroenterology and Hepatology Saitama Endoscopy and Liver Clinic Saitama Japan; ^3^ Department of Internal Medicine Division of Gastroenterology and Hepatology The Jikei University Kashiwa Hospital Chiba Japan

**Keywords:** chronic colitis, disease progression, diverticular disease, mesalamine, ulcerative colitis

## Abstract

**Objectives:**

Segmental colitis associated with diverticulosis (SCAD) has close endoscopic and pathological similarities to ulcerative colitis (UC) and Crohn's disease. Clinical data on SCAD are limited in Japan. We examined the endoscopic and clinicopathological features of patients with SCAD.

**Methods:**

This single‐center retrospective study included 13 patients with SCAD between 2012 and 2022. Endoscopic findings were categorized as follows: type A (swollen red patches 5–10 mm at the top of mucosal folds), mild and moderate type B (mild‐to‐moderate UC‐like findings), type C (aphthous ulcers resembling Crohn's disease), and type D (severe UC‐like findings).

**Results:**

Overall, six, five, and two patients were diagnosed with type A, mild type B, and moderate type B disease, respectively. Among the type A cases, two spontaneously progressed to moderate type B and one escalated to type D, necessitating an emergency sigmoidectomy owing to perforation peritonitis, despite repeated antibiotic treatments. Histopathologically, diffuse neutrophil and lymphocyte infiltration with cryptitis were noted in all type A cases, whereas UC‐like alterations were observed in type B and D cases. Seven type B cases were treated with oral 5‐aminosalicylic acid and/or salazosulfapyridine. Clinical remission was achieved in three mild type B cases and one moderate type B case, while clinical relapse and remission were noted in three moderate type B cases. No anti‐inflammatory treatment was required in three type A and two mild type B cases.

**Conclusions:**

Aggressive anti‐inflammatory treatment should be considered for SCAD with UC‐like findings due to the potential risk of severe ulceration, stenosis, and/or perforation.

## INTRODUCTION

In recent years, the incidence of diverticular diseases has increased in Japan because of the aging population.^1^ Most patients with colonic diverticulosis remain entirely asymptomatic, and the cumulative incidence of bleeding and diverticulitis from diverticulosis is 10% and 10%–25%, respectively.^2^, ^3^ Diverticulum‐associated complications include pathological conditions involving chronic inflammation of the colonic mucosa, known as segmental colitis associated with diverticulosis (SCAD), regardless of the presence of diverticulitis. SCAD is a chronic inflammatory disease mainly confined to the interdiverticular mucosa of the sigmoid colon, regardless of the inflammation affecting the diverticular orifices. Although SCAD can be difficult to distinguish from inflammatory bowel disease (IBD) because both have gastrointestinal symptoms and endoscopic findings such as diffuse loss of vascular pattern and pinpoint erosions, inflammation is not detected in the rectum either endoscopically or pathologically in patients with SCAD.^4^ Some studies have reported that SCAD may develop into ulcerative colitis (UC) with rectal involvement and severe colonic stenosis, resulting in the need for emergency surgeries.^5^, ^6^, ^7^, ^8^

In this case series, we retrospectively analyzed patients with SCAD to investigate the endoscopic and clinicopathological features and factors influencing disease progression.

## METHODS

We retrospectively reviewed the data of 13 patients diagnosed with SCAD by colonoscopy who presented to our institution between September 2012 and December 2022. Informed consent was obtained from all patients included in this study. The study protocol was approved by the Ethics Committee of the Onaka‐kai Onaka Clinic, and the study was conducted in accordance with the Declaration of Helsinki.

The final diagnosis of SCAD was established on the basis of a combination of endoscopic and histopathological features. Endoscopic findings included swollen red patches, erosion, loss of vascular pattern, erythema, hyperemia, and rectal sparing. Histopathological features included acute‐on‐chronic inflammation, such as cryptitis, crypt abscess, goblet cell depletion, basal plasmacytosis, and crypt architectural distortion. All patients were confirmed to have undergone rectal sparing via biopsy. The exclusion criteria were a history of IBDs (including those associated with primary sclerosing cholangitis [PSC]), ischemic colitis, infectious colitis, and the use of nonsteroidal anti‐inflammatory drugs. The endoscopic classification by identifying four different subtypes was established on the basis of a study by Tursi et al.: type A, “red round lesions measuring 5–10 mm at the top of mucosal folds”; type B, “diffuse loss of vascular pattern, edema, hyperemia, and pinpoint erosion resembling mild‐to‐moderate UC”; type C, “isolated aphthous ulcers resembling Crohn's disease”; and type D, “similar to type B but more severe with diffuse ulceration and reduced caliber of lumen”.^9^ Furthermore, based on the Mayo endoscopic subscore (MES),^10^ we subdivided type B into two categories: mild type B (MES 1‐like), characterized by “reduced vascular pattern, erythema, and mild friability,” and moderate type B (MES 2‐like), characterized by “diffuse absence of vascular pattern, marked erythema, friability, and erosions” (Figure S1).

## RESULTS

Table 1 presents the clinical characteristics of the patients. The patients comprised 10 men and 3 women, with ages ranging from 35 to 67 years (median: 52.2 years). Eight patients had a body mass index of >25 kg/m^2^, eight patients consumed alcohol (40 g ethanol/day), and nine patients were active smokers at the time of diagnosis. The underlying diseases included hypertension and dyslipidemia in six patients and post‐renal plantation chronic kidney disease in one patient. Before diagnosis, three patients had a history of acute diverticulitis for at least 3 years. Abdominal pain and hematochezia were observed in nine patients. Abdominal bloating and diarrhea were present in one patient each. Two patients were asymptomatic, and SCAD was confirmed during routine colonoscopy after a positive fecal occult blood test result.

**TABLE 1:** Clinical features of patients with segmental colitis associated with diverticulosis.

	No. of patients (%)
Age (mean ± SD) (years)	52.2 ± 7.7
Male sex	10 (76.9)
Overweight (BMI >25 kg/m^2)^	8 (61.5)
Alcohol consumption (>40 g of ethanol/day)	8 (61.5)
Active smoking at diagnosis	9 (69.2)
Underlying diseases	
Hypertension and dyslipidemia	6 (46.2)
Chronic kidney disease (IgA nephropathy)	1 (7.7)
History of acute diverticulitis	3 (23.1)
Symptoms	
Abdominal pain	5 (38.5)
Hematochezia	2 (15.4)
Abdominal pain and hematochezia	2 (15.4)
Asymptomatic (positive fecal occult blood test)	2 (15.4)
Abdominal bloating	1 (7.7)
Diarrhea	1 (7.7)

Abbreviations: BMI, Body mass index; IgA, immunoglobulin A; SD, Standard deviation.

Colonoscopic and histopathological findings are presented in Table 2. Endoscopic abnormalities were confined to the left colon in 10 patients. The endoscopic features of SCAD at diagnosis were classified into the following patterns: type A (*n* = 3 patients), mild type B (*n* = 5), moderate type B (*n* = 2), type A progression to moderate type B (*n* = 2), and type A progression to type D (*n* = 1). Histopathologically, all type A cases had diffuse infiltration of neutrophils and lymphocytes with cryptitis and no architectural changes in the crypts. In contrast, types B and D showed goblet cell depletion, crypt abscesses, and basal plasmacytosis with crypt architectural distortion.

**TABLE 2:** Colonoscopic and histopathological findings in patients with segmental colitis associated with diverticulosis.

				Histopathology			
Case	Colonoscopy Classification	Location	Extent of diverticula^†^	Cryptitis	Crypt abscess	Goblet cell depletion	Basal plasmacytosis
1	Type A	D	Mild	+	−	−	−
2	Type A	S	Mild	+	−	−	−
3	Type A	T	Mild	+	−	−	−
4	Mild‐type B	A, S	Moderate	+	+	+	+
5	Mild‐type B	A	Mild	+	−	+	−
6	Mild‐type B	S	Mild	+	−	+	+
7	Mild‐type B	C	Mild	+	−	+	−
8	Mild‐type B	D	Mild	+	+	+	+
9	Moderate‐type B	C, A	Moderate	+	+	+	+
10	Moderate‐type B	S	Mild	+	+	+	−
11	Type A→moderate type B	S	Mild	+→+	−→+	−→+	−→+
12	Type A→moderate type B	S	Mild	+→+	−→+	−→+	−→+
13	Type A→type D	S	Moderate	+→+	−→+	−→+	−→+

*Note*: A: ascending colon, C: Cecum, D: Descending colon, S: Sigmoid colon, T: Transverse colon.

Type A: red round lesions measuring 5–10 mm at the top of mucosal folds. Mild‐type B: educed vascular pattern, erythema, and mild friability resembling mild ulcerative colitis. Moderate‐type B: Diffuse absence of vascular pattern, marked erythema, friability, and erosions resembling moderate ulcerative colitis. Type D: Diffuse ulceration and reduced lumen caliber resembling severe ulcerative colitis.

^†^ The extent of diverticula was defined as follows: mild, located in one colon segment; moderate, located in two colon segments; severe, located in three or more colon segments.

Laboratory results, performed in all patients at diagnosis, showed a high inflammatory response in only one case progressing from type A to type D (WBC count: 10,400 cells/µg, CRP level: 3.00 mg/dL). No patients showed elevated hepatobiliary enzyme levels. Computed tomography and ultrasonography revealed thickening of the colonic wall in four cases of moderate type B and one case of type D, all of which had diverticula in the colon segment. However, no patient exhibited dilatation of intraextrahepatic ducts and thickening of the bile duct wall.

**FIGURE 1:**
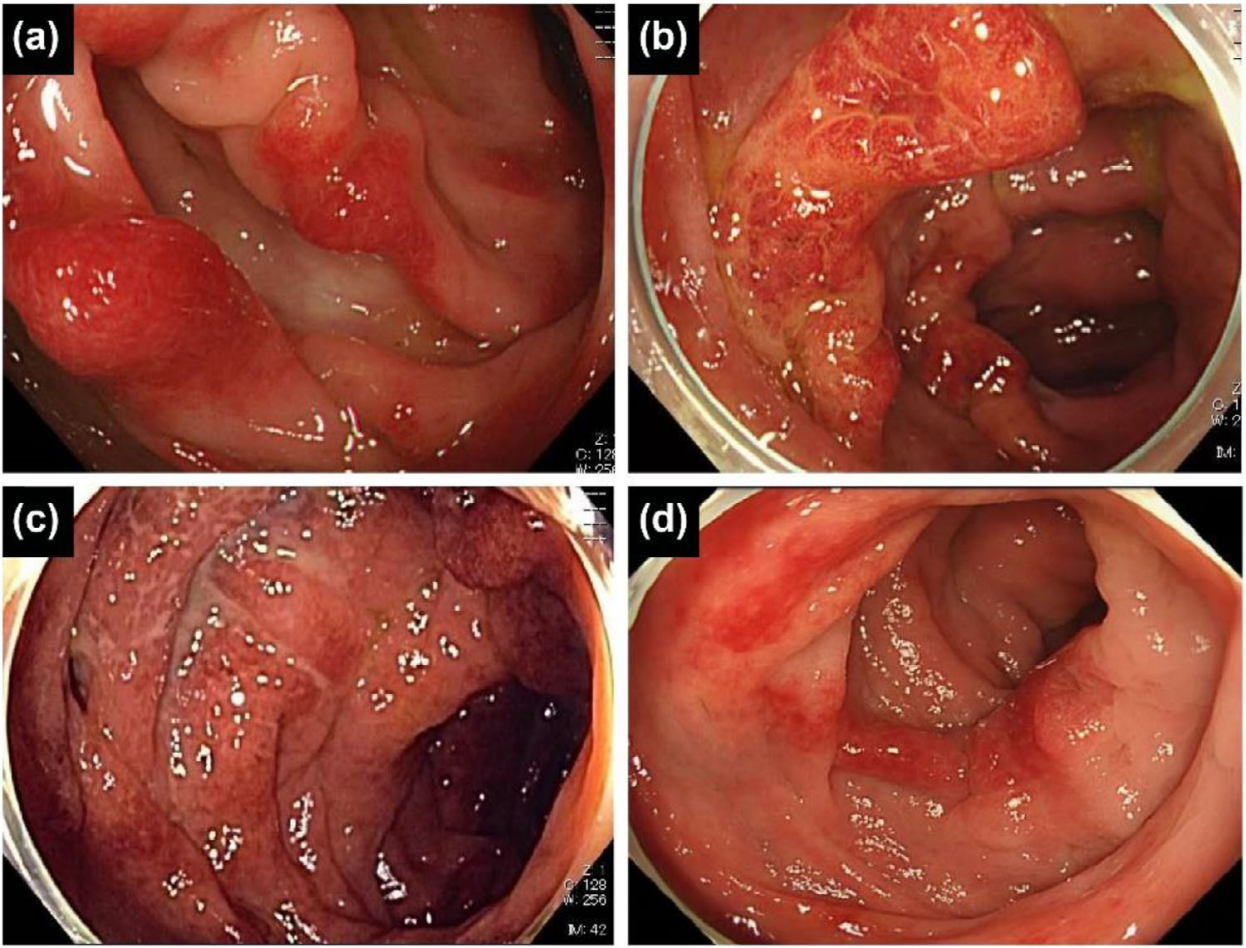
Case 12 is a 51‐year‐old man. (a) Colonoscopy shows extensive edematous erythema, equivalent to Tursi's classification type A, in the interdiverticular mucosa of the sigmoid colon at diagnosis. (b) Extensive edematous erythema was enlarged at 3 years after initial diagnosis. (c) Diffuse absence of normal vascularity and friable erythematous and edematous mucosa with mucous exudate, equivalent to moderate type B were observed at 4 years after initial diagnosis. (d) Endoscopic findings show improvement from moderate type B to type A after 6 months of treatment with 5‐ASA. 5‐ASA: 5‐aminosalicylic acid.

**FIGURE 2:**
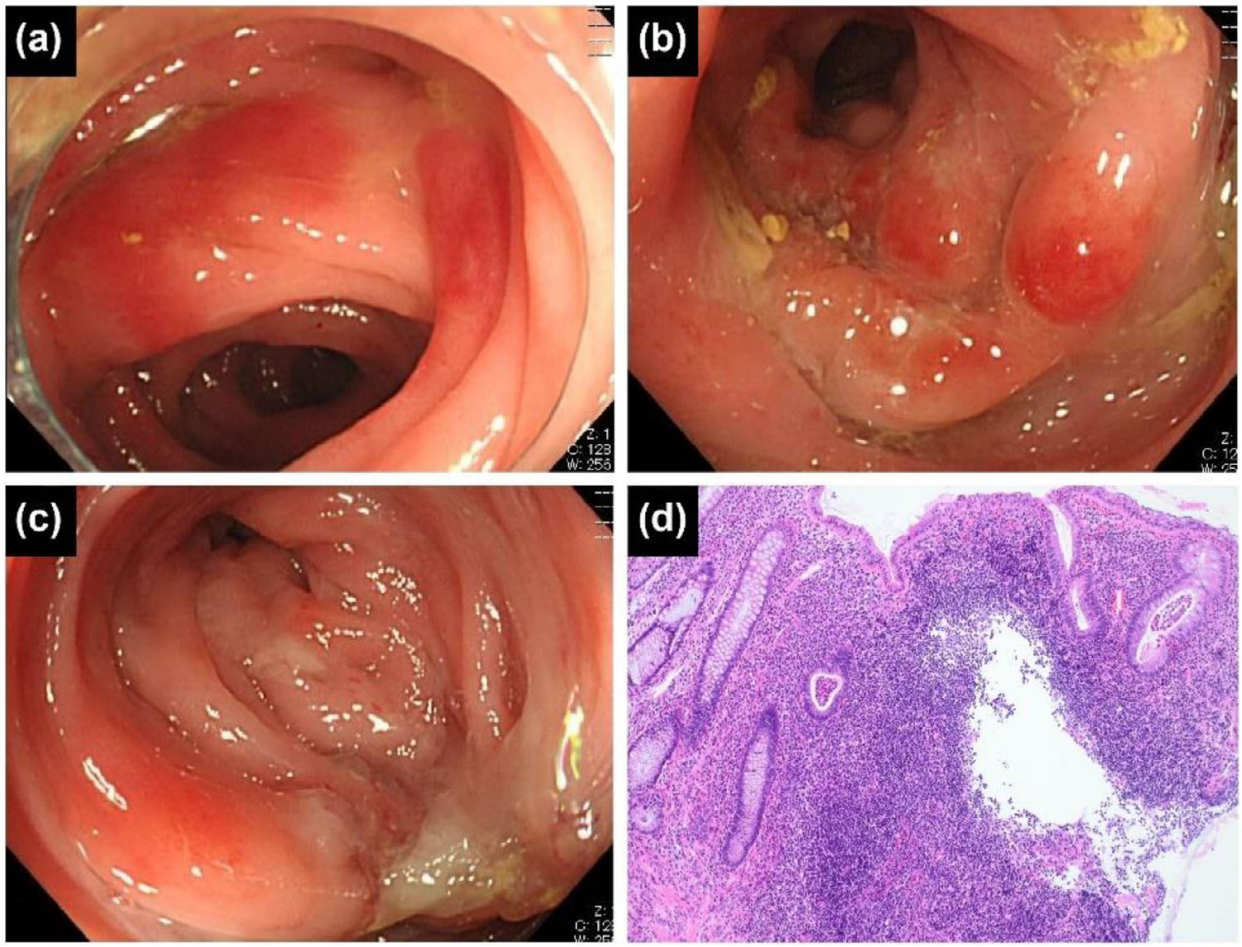
Case 13 is a 48‐year‐old man. (a) Colonoscopy shows marked edematous erythema, equivalent to Tursi's classification type A, between the sigmoid diverticula at diagnosis. (b) After 2 years of repeated antibiotic treatment with levofloxacin (500 mg/day), colonoscopy revealed polypoid lesions with marked erythema. (c) Four years after the initial diagnosis, diffuse ulceration and reduced caliber of the lumen, equivalent to Tursi's classification type D, were observed. (d) A biopsy specimen obtained from type D lesions showed cryptitis, crypt abscess, goblet cell depletion, and basal plasmacytosis, with crypt architectural distortion and diffused infiltration of neutrophils and lymphocytes into the lamina propria.

**FIGURE 3:**
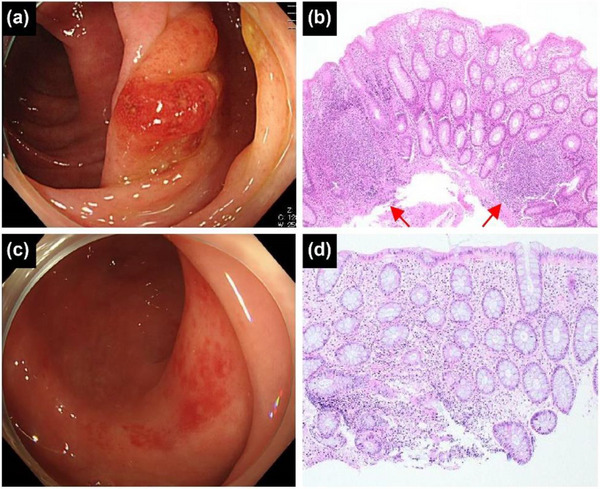
Comparison of progression to UC‐like findings and spontaneous remission in patients with type A. (a) Colonoscopic image shows polypoid lesions with marked erythema, and (b) histopathological image shows lymphoid follicular hyperplasias (red arrows) in patients with UC‐like findings. (c) Colonoscopic and (d) histopathological images show neither of the above findings in patients with spontaneous remission. UC: ulcerative colitis.

The treatment and clinical course of the patients are presented in Table 3. The median follow‐up period was 12 months (interquartile range: 6–28 months). Seven patients with type B disease received oral 5‐aminosalicylic acid (5‐ASA) and/or salazosulfapyridine (SASP) at various doses (3000–4800 mg/day); among them, four patients maintained clinical remission, and the remaining three patients with moderate type B disease experienced clinical relapse and remission for years. Five patients (three type A and two mild type B cases) did not require anti‐inflammatory treatment, including 5‐ASA, and achieved endoscopic and/or clinical remission. Two patients with type A disease (cases 11 and 12) spontaneously progressed to moderate type B at 3–4 years after the initial diagnosis, and after 6 months of 5‐ASA treatment, their colonoscopic findings demonstrated improvement from moderate type B to type A (Figure 1). In one patient with type A disease (case 13), a colonoscopy revealed marked edematous erythema—equivalent to type A, localized to crescent‐shaped mucosal folds at the initial endoscopy—and polypoid lesions with marked erythema, after 2 years of repeated antibiotic treatment with levofloxacin (500 mg/day). Four years after the initial diagnosis, diffuse ulceration and reduced lumen caliber, equivalent to type D, were observed. Eventually, emergency sigmoidectomy was performed for perforation peritonitis at 3 months after the diagnosis of type D disease. No postoperative recurrence was observed during the 5 years of follow‐up (Figure 2). When comparing the progression to type B or D and spontaneous remission in patients with type A disease, the following findings were observed on previous colonoscopic and histopathological examinations: all patients with progression to UC‐like findings exhibited erythematous polypoid lesions and lymphoid follicular hyperplasia; in contrast, neither of these findings was observed in patients with spontaneous remission (Figure 3).

**TABLE 3:** Treatment and clinical course of patients with segmental colitis associated with diverticulosis.

Case	Classification	Treatment	Endoscopic findings after induction	Clinical course	Duration of follow‐up (months)
1	Type A	Probiotics	N/A	Remission	3
2	Type A	Probiotics	N/A	Remission	3
3	Type A	None	Spontaneous remission	Remission	12
4	Mild‐type B	None	Spontaneous remission	Asymptomatic	6
5	Mild‐type B	None	Spontaneous remission	Asymptomatic	6
6	Mild‐type B	Oral 5‐ASA	Remission	Remission	28
7	Mild‐type B	Oral 5‐ASA	Remission	Remission	10
8	Mild‐type B	Oral 5‐ASA	N/A	Remission	12
9	Moderate‐type B	Oral 5‐ASA	Remission	Remission	15
10	Moderate‐type B	SASP	Response (type A)	Remission and relapse	21
11	Type A→Moderate type B	Oral 5‐ASA	Response (type A)	Remission and relapse	41
12	Type A→Moderate type B	Oral 5‐ASA	Response (type A)	Remission and relapse	60
13	Type A	Antibiotics (LVFX)	Progression (type D)	Perforation→Surgery	66

Abbreviations: 5‐ASA, 5‐aminosalicylic acid; LVFX, levofloxacin; N/A, not applicable; SASP, salazosulfapyridine.

Type A: Red round lesions measuring 5–10 mm at the top of mucosal folds. Mild‐type B: Reduced vascular pattern, erythema, and mild friability resembling mild ulcerative colitis. Moderate‐type B: Diffuse absence of vascular pattern, marked erythema, friability, and erosions resembling moderate ulcerative colitis. Type D: Diffuse ulceration and reduced lumen caliber resembling severe ulcerative colitis.

## DISCUSSION

SCAD was first described in the early 1980s as a type of colitis characterized by sigmoid diverticulosis with patchy or diffuse mucosal inflammation.^11^, ^12^ Currently, there is limited understanding of the pathogenesis, incidence, treatment, and prognosis of this disease. The frequency of SCAD reportedly ranges from 1.9% to 11% in patients with diverticulosis and varies by cohort.^9^, ^13^, ^14^ SCAD also occurs more frequently in middle‐aged and elderly men,^13^, ^14^ with a median age at diagnosis of 52.2 years and a male prevalence of 76.9%.

The etiology of SCAD has not yet been fully understood; however, several studies have suggested that it is likely multifactorial and associated with diverticula. These diverticula can lead to mucosal prolapse, which can cause ischemia. Additionally, fecal stasis and subsequent microbial dysbiosis can occur, which may stimulate an immune response.^8^, ^15^, ^16^, ^17^ With regard to alterations in the intestinal microbiota in colonic diverticula, Barbara et al. reported that patients with diverticula demonstrated reduced levels of bacterial species, such as *Clostridium* cluster IX, that produce short‐chain fatty acids with anti‐inflammatory effects.^18^ In a study by Tursi et al., expression of tumor necrosis factor‐alpha in patients with SCAD was associated with the severity of endoscopic damage, similar to IBD, supporting the hypothesis that SCAD may be a part of the IBD spectrum.^19^ Indeed, Vulsteke et al. reported that progression to typical UC was observed in three of 37 (8.11%) patients with SCAD, with a follow‐up period of 9–18 months before UC diagnosis.^20^

Patients with SCAD present with diverse clinical symptoms.^9^ Among our patient cohort, lower abdominal pain was the most frequent symptom (*n* = 7 patients), followed by intermittent hematochezia (*n* = 4), no symptoms (*n* = 2), diarrhea (*n* = 1), and abdominal bloating (*n* = 1). The clinical presentation of SCAD is very similar to that of IBD, whereas systemic symptoms such as fatigue, fever, and weight loss are rare in patients with SCAD.^21^ In some cases, SCAD can be detected incidentally during routine colonoscopy for colon cancer screening.^20^

Endoscopic findings of SCAD have various characteristics, ranging from edema and erythema between the colonic diverticula and erosion to mucosal friability, and ulceration, resembling UC. Therefore, endoscopically differentiating SCAD from other forms of colitis, such as IBD, ischemic colitis, infectious colitis, and drug‐induced colitis, may be difficult. In particular, as colonic inflammation in SCAD can mimic IBD, to make an accurate differential diagnosis, the presence of localized inflammation between the colonic diverticula and rectal sparing should be confirmed endoscopically and histopathologically.^22^ The high prevalence of rectal sparing in IBD associated with PSC has been reported. Specifically, the prevalence of rectal sparing in IBD associated with PSC was 52%, compared to only 6% in typical chronic UC (*p* < 0.001).^23^ Therefore, it is important to conduct laboratory and imaging examinations to rule out the presence of PSC. Tursi et al. classified endoscopic features into four subtypes and revealed a significant association between histopathological findings and endoscopic severity,^9^ consistent with our findings.

In contrast to the relapsing and remitting course of IBD, SCAD has a favorable clinical course, and spontaneous remission can be expected. However, some patients may experience persistent colonic inflammation and relapse, leading to the use of 5‐ASA, steroids, and/or surgery.^24^ In a follow‐up study of the clinical outcomes of SCAD according to subtypes, patients with type A and C disease often had a benign clinical course, whereas those with type B and D disease had a higher propensity for relapse and remission.^25^ Aggressive treatment and endoscopic follow‐up should be considered with caution in patients with type B and D disease, although optimal treatment strategies and endoscopic surveillance intervals have not yet been established. Vulsteke et al. suggested that oral administration of 5‐ASA should be chosen as the first‐line therapy because it has a proven remission rate of 88%, whereas antibiotic therapy was not very effective with a remission rate of about 10%.^20^ In a recent translational study using healthy mice,^26^ oral administration of 5‐ASA increased the population of short‐chain fatty acids‐producing bacteria, which may have contributed to the suppression of mucosal inflammation. This finding may be associated with a higher success rate of 5‐ASA therapy for SCAD.

In the present study, three of five patients with mild type B disease (MES1‐like findings) achieved symptomatic improvement within 1 month of initiating oral 5‐ASA and did not require maintenance therapy; the remaining two patients were left untreated without any symptoms and had normal colonoscopic findings at 3 months after diagnosis. In contrast, four patients with moderate type B disease (MES2‐like findings) required maintenance therapy with oral 5‐ASA and/or SASP over a 1‐year period. Of these patients, three could not achieve complete endoscopic remission and experienced repeated clinical relapse and remission despite continuing the use of oral 5‐ASA and/or SASP. However, they managed to maintain clinical remission by increasing the doses of 5‐ASA and SASP to the maximum level during relapse. The treatment algorithm for SCAD proposed by Kucejko et al. recommends a combination of probiotics for 6 weeks and corticosteroids with reduced systemic side effects for 4 weeks in patients who experience relapse despite using 5‐ASA. Tumor necrosis factor‐alpha inhibitors and surgery are recommended in cases of corticosteroid dependence.^16^ Therefore, we believe that different treatment approaches, including corticosteroids, should be considered for patients with type B SCAD refractory to 5‐ASA.

Among the six patients with type A disease, erythematous polypoid lesions with histopathological lymphoid follicular hyperplasia (LFH) were observed in three patients with progression to type B or D disease. Our results suggest that both endoscopic and histopathological assessments may be useful for predicting the progression from type A to UC‐like findings. To the best of our knowledge, no previous study has investigated the natural progression and clinical significance of LFH in relation to SCAD. Hong et al. reported that LFH may reflect a dysregulated immune response to unknown environmental triggers and maybe the initial form of UC.^27^ Further cases with careful follow‐up are required to elucidate the progression of endoscopic and histopathological features in SCAD type A.

This study had some limitations. First, this was a single‐center, retrospective study, with a limited sample size, which may have resulted in selection bias. Second, endoscopic evaluation after treatment was not performed in all patients with SCAD at our institution. Third, in some patients, the surveillance period was not sufficient to analyze long‐term outcomes, including relapse and remission.

In conclusion, our retrospective study suggests that most cases of SCAD have favorable prognoses; however, in a subset of patients with UC‐like findings, aggressive anti‐inflammatory treatment should be considered due to the potential to cause severe ulceration, stenosis, and/or perforation. As previously reported, oral 5‐ASA therapy may generally be effective for SCAD. However, further prospective studies should be conducted with a larger number of patients, including 5‐ASA refractory and recurrent cases.

## CONFLICT OF INTEREST STATEMENT

None.

## Supporting information


**Figure S1** Endoscopic classification of segmental colitis associated with diverticulosis.
