# Supplementary figures and images for: Endoscopic and clinicopathological features of segmental colitis associated with diverticulosis
Source: DEN Open. 2024 Apr 2;4(1):e356. doi: 10.1002/deo2.356 (PMC10987790; doi:10.1002/deo2.356)

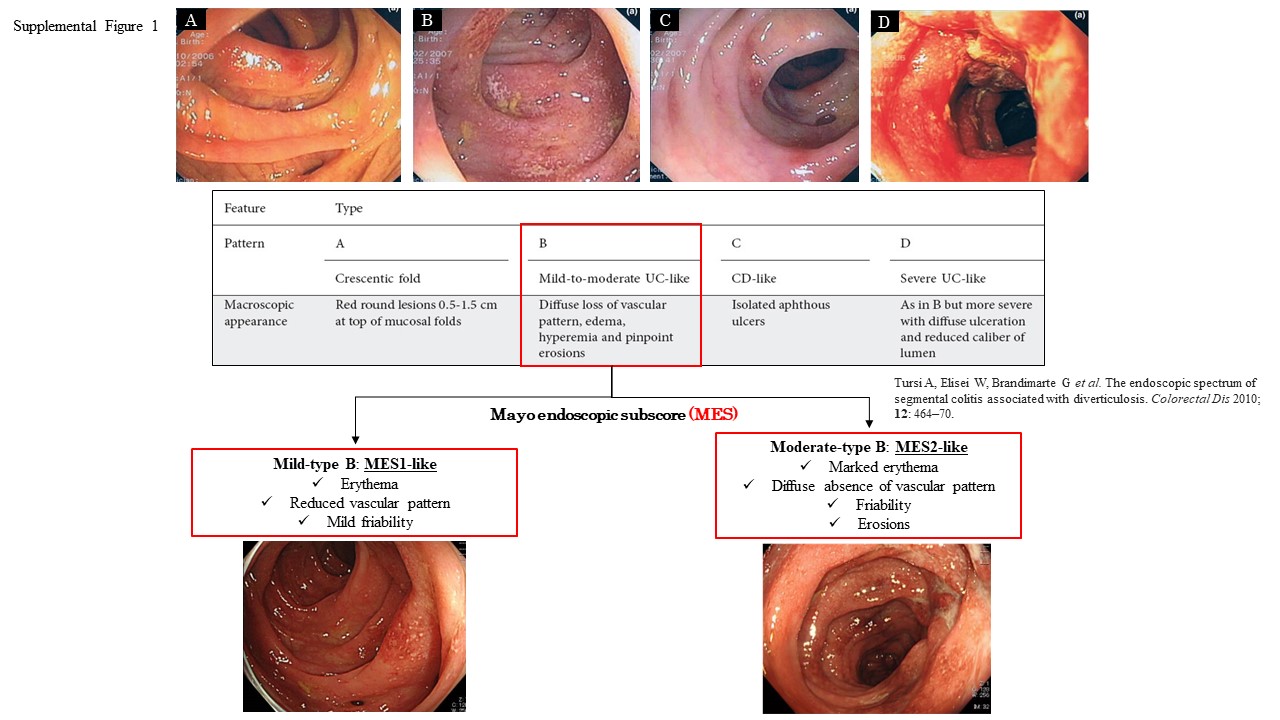

Supplement: Supplementary file 1 — Figure S1 Endoscopic classification of segmental colitis associated with diverticulosis. [file DEO2-4-e356-s001.jpg]
